# Phytochemical Characterization and In Vitro Anti-Inflammatory, Antioxidant and Antimicrobial Activity of *Combretum Collinum* Fresen Leaves Extracts from Benin

**DOI:** 10.3390/molecules25020288

**Published:** 2020-01-10

**Authors:** Peter Marquardt, Rick Seide, Cica Vissiennon, Andreas Schubert, Claudia Birkemeyer, Virgile Ahyi, Karin Fester

**Affiliations:** 1 Institute of Pharmacy, Leipzig University, Johannisallee 21-23, 04103 Leipzig, Germany; rick.seide@web.de (R.S.); karin.fester@uni-leipzig.de (K.F.); 2 Institute for Medical Physics and Biophysics, Leipzig University, Härtelstr 16-18, 04107 Leipzig, Germany; cica.vissiennon@uni-leipzig.de; 3 Antimicrobial Agents Unit, Fraunhofer Institute for Cell Therapy and Immunology, Perlickstraße 1, 04103 Leipzig, Germany; andreas.schubert@izi.fraunhofer.de; 4 Institute of Analytical Chemistry, Leipzig University, Linnéstr 3, 04103 Leipzig, Germany; birkemeyer@chemie.uni-leipzig.de; 5 Inter-Regional University of Industrial Engineering Biotechnologies and Applied Sciences, IRGIB Africa University, Cotonou 07 BP 231, Benin; ahyivirgile@yahoo.fr; 6 Faculty of Natural and Environmental Sciences, Zittau/Görlitz University of Applied Sciences, 02763 Zittau, Germany

**Keywords:** *Combretum collinum*, anti-inflammatory, antimicrobial, keratinocytes, HaCaT

## Abstract

Leaves from *Combretum collinum* Fresen (Combretaceae) are commonly used for the treatment of inflammatory conditions, wound healing and bacterial infections in traditional West African medicine. This research focuses on the characterization of the phenolic profile and lipophilic compounds of leaves extracts of *C. collinum*. Studies of the in vitro anti-inflammatory activity were performed in TNFα stimulated HaCaT cells and antibacterial activity was evaluated with agar well diffusion and microdilution assays. Antioxidant activity was determined by DPPH and ABTS assays and compared to standards. The phytochemical studies confirmed myricetin-3-*O*-rhamnoside and myricetin-3-*O*-glucoside as major components of the leaves extracts, each contributing significantly to the antioxidant activity of the hydrophilic extracts. GC-MS analysis identified 19 substances that were confirmed by comparison with spectral library data and authentic standards. *Combretum collinum* aqueous leaves extract decreased pro-inflammatory mediators in TNFα stimulated HaCaT cells. Further investigations showed that myricetin-3-*O*-rhamnoside has an anti-inflammatory effect on IL-8 secretion. In the antimicrobial screening, the largest inhibition zones were found against *S. epidermidis*, *MRSA* and *S. aureus*. MIC values resulted in 275.0 µg/mL for *S. epidermidis* and 385.5 µg/mL for *MRSA*. The in vitro anti-inflammatory, antibacterial and antioxidant activity supports topical use of *C. collinum* leaves extracts in traditional West African medicine.

## 1. Introduction

Sub-Saharan Africa is a region of the world where the use of traditional herbal medicines has long been widespread, and a large part of the population depends on it to maintain their health [1]. Particularly in rural areas with insufficient access to modern medicine, such as Northern Benin, a remarkably high number of species is used as herbal remedies [2]. In this region, combinations of various medicinal plants are used externally for the treatment of inflammation, fractures and related infections. For example, a mixture of *Piliostigma thonningii* (Schum.) Milne-Redh., *Ficus thonningii* Blume, *Fadogia agrestis* Schweinf. Ex Hiern, *Entada Africana* Guill. and Perr., *Chasmanthera dependens* Hochst., and *Combretum collinum* Fresen is used traditionally for the treatment of musculoskeletal disorders and pain and has already shown anti-inflammatory effects in a preliminary clinical observation [3]. *Combretum collinum* Fresen (Combretaceae) as an important ingredient of this traditional herbal combination is a small, semi-evergreen tree or shrub, that is native to West and South Africa [4], and is extensively used in traditional West African medicine. The leaves of the plant are used for treating wounds and ulcers and are also applied as ear drops to treat earache. Compresses or baths with crushed leaves are used to treat inflammatory diseases such as rheumatism [5]. The leaves are also used to cure diseases associated with microbial infection: Leaves infusions of *C. collinum* are generally used to relieve gastrointestinal problems such as diarrhea and ascariasis, but also to treat lung problems like cough and bronchitis [6]. In some in vitro tests, pharmacological effects have already been demonstrated for *C. collinum* such as antibacterial activity against Pseudomonas and effects on tumor growth and development [7]. *Combretum collinum* has already been reported to contain stilbenoids (combretastatins) and phenanthrenes [8]. Compared to other *Combretum* species, however, the components of *C. collinum* are still relatively under-researched and only a few individual compounds such as mollic acid and its glycosides have been identified [9]. Knowledge about the underlying pharmacological mechanisms of actions of *C. collinum* leaves extracts is still scarce. Therefore, this research focuses on the characterization of the phenolic profile and lipophilic compounds of leaves extracts of *C. collinum* from Benin. We studied the in vitro anti-inflammatory effect of *C. collinum* leaves extract and its major phenolic compounds in immortalized human keratinocytes (HaCaT) to determine whether its use against inflammatory diseases can be supported. Since inflammatory conditions are closely related to oxidative stress, the antioxidant effect of the extract and its major compounds was evaluated. Furthermore, in vitro tests were performed to characterize the spectrum of antibacterial action of *C. collinum* extract in order to investigate whether its application in infections is justified.

## 2. Results and Discussion

### 2.1. Structure Elucidation of Compounds in the 50% Ethanol Extract from C. collinum Leaves and Phytochemical Analysis of the n-Hexane Extract

**Figure 1:**
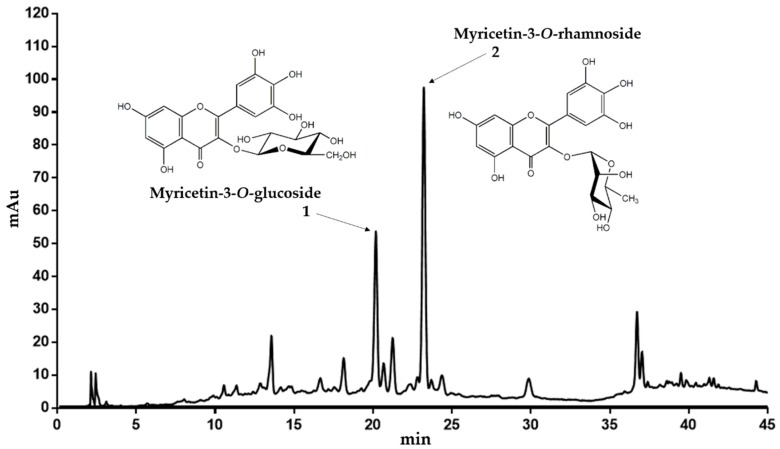
Representative HPLC chromatogram of the EtOH 50% extract of *C. collinum* (5 mg/mL extract dissolved in ACN 5%, injection volume 20 µL, detection wavelength = 320 nm). The main compounds could be confirmed by LC-MS experiments, ^1^H-NMR and spiking experiments.

Dried leaves of *C. collinum* (600 g) were degreased with petroleum ether and the drug residue was extracted with 50% (*v*/*v*) ethanol (EtOH, extract yield 14.1% relative to degreased plant material). The obtained EtOH 50% extract was then partitioned between ethyl acetate (EtOAc phase: yield 13.9% related to EtOH 50% extract) and water (water phase: yield 44% related to EtOH 50% extract). Thin layer chromatography (TLC) analysis indicated that the ethyl acetate extract was enriched in flavonoids after spraying with natural product reagent A. Thus, a high-performance liquid chromatography (HPLC) fingerprint analyses of the EtOH 50% extract and the EtOAc extract revealed two main peaks with flavonoid UV-spectra, which were enriched in the EtOAc extract. The EtOH 50% and EtOAc extracts were subjected to liquid chromatography-electrospray ionization-mass spectrometry (LC-ESI-MS) analysis. Subsequently, the recorded total ion current trace (TIC) in positive and negative mode was then compared with the UV chromatogram at 320 nm and the masses of the most important peaks were analyzed (Supplementary Material). Since ESI-MS is a soft ionization technique, it provides a significant amount of structural information for phenolic glycosides. The ESI-MS spectra of flavonol glycosides in the positive mode generally have the main peak corresponding to the aglycon [A + H]^+^ ion and a weak peak corresponding to the glycoside [M + H]^+^ pseudo molecular ion. The mass difference between [M + H]^+^ and [A + H]^+^ ions gives useful information about the type of sugar. In the LC-ESI-MS analyses of the EtOH 50% and the EtOAc extracts, the two flavonoid peaks **1** and **2** revealed fragmentation patterns specific for glycosides (Table 1). As shown for **1**, the signal at *m*/*z* 481 corresponded to the [M + H]^+^ pseudo molecular ion, while [A + H]^+^ at *m*/*z* 319 was attributed to the aglycone. While the neutral loss of 162 indicated a hexose unit, the aglycone was tentatively assigned as myricetin due to its mass (Mr 318). In negative mode, peak **1** showed a [M − H]^−^ pseudo molecular ion at *m*/*z* 479. Since myricetin-3-*O*-glucoside was already described in *Combretum micranthum* [10], we concluded that *C. collinum* might also contain myricetin-3-*O*-glucoside (peak **1** of the HPLC chromatogram, Figure 1). For peak **2**, ions were observed at *m*/*z* 465 and *m*/*z* 319 (Table 1). The ion at *m*/*z* 465 corresponded to the [M + H]^+^ pseudo molecular ion. The signal at *m*/*z* 319 was tentatively assigned to the aglycone (myricetin), which had been generated by the loss of a hexosyl unit in the form of a deoxy-sugar (146 amu). In negative mode, peak **2** showed a [M − H]^−^ pseudo molecular ion at *m*/*z* 463. Therefore, myricetin-3-*O*-rhamnoside was suggested as compound **2** (Figure 1).

To confirm these assumptions further, the EtOAc extract was chromatographed on Sephadex LH-20 with a gradient of 96% ethanol. Individual fractions were then combined due to their similar TLC and HPLC profile and recrystallized in ethyl acetate. Combined fractions F31–F33 provided satisfactory purity for ^1^H-NMR analysis. Comparison of the ^1^H-NMR spectroscopic data of the compound revealed a typical flavonol pattern of a myricetin aglycon (Table 2). Further HPLC experiments with reference substances and spiking experiments subsequently confirmed both, the myricetin-3-*O*-glucoside and myricetin-3-*O*-rhamnoside as the main flavonol constituents of the EtOH 50% and EtOAc extracts of *C. collinum.*

**Figure 2:**
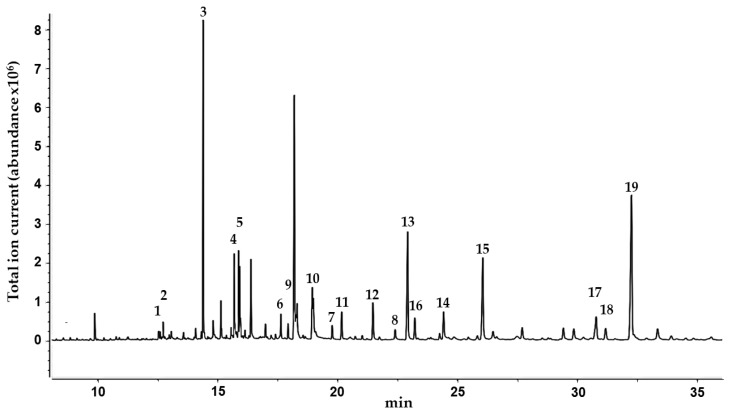
GC-MS chromatogram of the *n*-hexane extract of the leaves of *C. collinum* after silylation with BSTFA. Peaks were identified with the NIST08 database.

In order to obtain further information on the lipophilic components of *C. collinum*, a GC-MS analysis of a silylated hexane extract of the leaves was performed (Figure 2). By comparison with the reference spectra of the NIST08 database, 19 compounds were identified, and their presence in the hexane extract was partially confirmed by GC-spiking experiments (Table 3). In agreement with earlier GC-MS studies of the related *Combretum* species *Combretum mucronatum,* our results revealed the presence of malic, myristic, palmitic, stearic, oleic and arachidonic acids, squalene and heptacosane [12].

### 2.2. In-Vitro Antioxidant Activity of C. collinum EtOH 50% Leaves Extract

**Figure 3:**
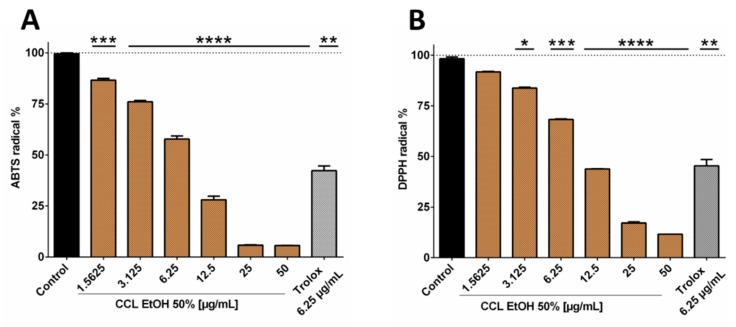

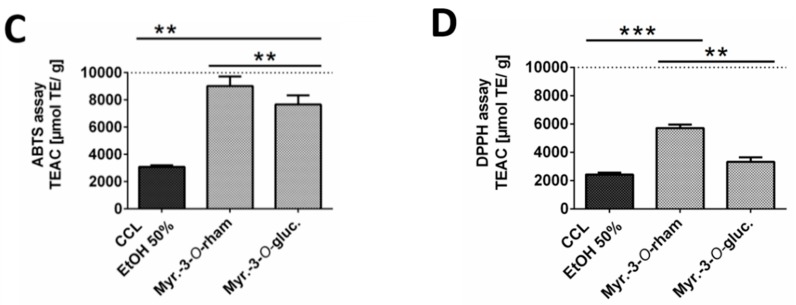
Antioxidant activity of 50% ethanolic extract of *C. collinum* extract, measured by ABTS (**A**) and DPPH assays (**B**). CCL EtOH 50% showed significant concentration-dependent antioxidant activity compared to control (pure ABTS or DPPH solution incubated with a blank). Trolox 6.25 µg/mL was used as a positive control. The two diagrams **C** and **D** show that the two main compounds of the extract had significantly higher antioxidant activity than CCL EtOH 50% in both the ABTS (**C**) and the DPPH assays (**D**) and thus most likely significantly contributed to the overall antioxidant activity of the extract; values in (**C**) and (**D**) are given in Trolox Equivalent Antioxidative Capacity (TEAC). *n* = 3 replicates, data presented as mean ± SEM, significant for * *p* ≤ 0.05, ** *p* ≤ 0.01, *** *p* ≤ 0.001, **** *p* ≤ 0.001 compared to the control in ordinary one-way ANOVA (analysis of variance).

Since the 50% ethanolic extract of *C. collinum* leaves (CCL EtOH 50%) had already shown pronounced activity in the DPPH-TLC test, it was of interest to quantify its antioxidant activity. CCL EtOH 50% was therefore investigated for its antioxidant effect using the ABTS and DPPH assays. Trolox at a concentration of 6.25 µg/mL was used as a positive control in both antioxidative tests. Figure 3B shows that CCL EtOH 50% exhibited antioxidant activity in concentrations of 1.56 µg/mL to 50 µg/mL compared to the control (pure DPPH solution incubated with blank) in the DPPH assay. The pure substances myricetin-3-*O*-rhamnoside and myricetin-3-*O*-glucoside were also tested for their antioxidant effect and demonstrated a higher antioxidant activity than the extract with values of 5715 ± 249 µmol TE/g for myricetin-3-*O*-rhamnoside and 3324 ± 322 µmol TE/g for myricetin-3-*O*-glucoside compared to CCL EtOH 50% with 2433 ± 129 µmol TE/g (Figure 3D). CCL EtOH 50% also showed distinct antioxidant activity of 3084 ± 121 µmol TE/g in the ABTS system (Figure 3A). For comparison, the antioxidant activity of myricetin-3-*O*-glucoside and myricetin-3-*O*-rhamnoside was determined to be 7667 ± 666 µmol TE/g and 9024 ± 700 µmol TE/g, respectively (Figure 3C). Trolox at a concentration of 6.25 µg/mL as positive control showed similar activity as the CCL EtOH 50% at 12.5 µg/mL, indicating that CCL EtOH 50% has a high antioxidant potential.

These results show that *C. collinum* leaves extract may act as a donor of H• radicals which bind to the DPPH radical forming hydrazine and also as an H• donor to ABTS radicals. Comparable values of extract and pure substances for the two methods are probably based on the fact that both methods are relying on similar mechanisms of single-electron transfers. It is known that the phenolic constituents of plant extracts are responsible for their antioxidant effect. Flavonoids, in particular, have pronounced antioxidant properties, since they contain OH-groups that act as H• donors and can, therefore, exhibit antioxidant effects [13]. The combination of a 3-hydroxy group with a 2,3-double bond enhances the resonance stabilization for the electron delocalization. In myricetin, the galloyl structure in the B-ring also plays a decisive role in the pronounced antioxidant activity [14]. The observed differences in antioxidant activities between myricetin-3-*O*-rhamnoside and myricetin-3-*O*-glucoside could be attributed to their different sugar moieties. Apart blocking free OH groups, which are crucial for hydrogen abstraction and free radical scavenging, the sugar substituent is capable of diminishing the coplanarity of the B-ring relative to the rest of the flavonoid and providing it with hydrophilicity [15]. Whether the sugar component is glucose, rutinose or rhamnose is also of significance. In comparison to rutinose, for example, a rhamnose group on quercetin significantly reduces the radical scavenging activity against radicals from stimulated human neutrophils [16]. HPLC quantification for myricetin-3-*O*-rhamnoside and myricetin-3-*O*-glucoside in the CCL EtOH 50% extract resulted in concentrations of 17.95% ± 0.76% and 6.06% ± 0.73%, supporting that both substances contribute significantly to the antioxidant potential of the extract. Since inflammatory diseases are strongly associated with oxidative stress, substances that can alleviate oxidative stress are of interest for the treatment of such diseases [17]. Consequently, the pharmacological effects of *C. collinum* leaves extracts on inflammation were investigated.

### 2.3. In-Vitro Anti-Inflammatory Activity of C. collinum Aqueous Leaves Extract, Myricetin-3-O-rhamnoside and Myricetin

**Figure 4:**
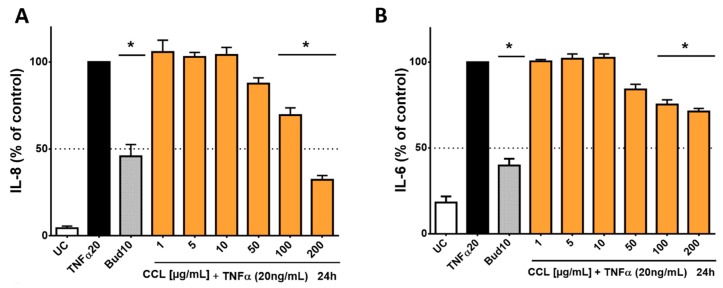
Influence of *C. collinum* aqueous leaves extract on IL-8- and IL-6-levels released by HaCaT cells after stimulation with TNFα (20 ng/mL): An aqueous leaves extract of *C. collinum* (CCL) concentration-dependently reduced the levels of IL-8 (**A**) and IL-6 (**B**) at nontoxic concentrations. CCL extract was tested in concentrations ranging from 1–200 µg/mL, resulting in an IC_50_ value of 142.5 µg/mL for IL-8 (**A**) and a decrease of IL-6 release by 29.3% for the highest extract concentration of 200 µg/mL respectively (**B**). 10 µM budesonide (Bud 10) was used as a positive control, UC = Untreated control, *n* = 4–6 replicates, data presented as mean ± SEM, significant for * *p* ≤ 0.05 compared to TNFα control in ordinary one-way ANOVA.

For the treatment of inflammatory conditions, aqueous extracts of *C. collinum* leaves are usually used, as is often the case in ethnomedical practice. Therefore, an aqueous extract (CCL) and not an ethanolic extract (CCL EtOH 50%; as studied in the phytochemical analysis) was used for the pharmacological investigations. In addition, the analysis and comparison of the aqueous and ethanolic extracts by HPLC experiments showed the same composition of the main components myricetin-3-*O*-rhamnoside and myricetin-3-*O*-glucoside. In our cell-based inflammation model, we chose an incubation scheme in which HaCaT cells, an immortalized keratinocyte cell line, was used. As a proof of concept, HaCaT cells were stimulated with TNFα and produced high levels of IL-8 and IL-6, which initially confirmed the function of the cell model. Budesonide, which was applied as positive control, reduced the release of IL-6 and IL-8 by 60.1% ± 4.7% respectively 54.3% ± 6.8%. CCL caused a concentration-dependent reduction of IL-6 and IL-8 levels compared to the overregulated release of IL-8 and IL-6 in the cell supernatant when treated only with TNFα (20 ng/mL). CCL was tested in concentrations ranging from 1–200 µg/mL, resulting in an IC_50_ value of 142.5 µg/mL for IL-8 and a decrease of IL-6 release by 29.3% for the highest extract concentration of 200 µg/mL respectively (Figure 4).

Subsequently, the cell viability of the HaCaT cells was tested by the MTT test. HaCaT cells were incubated with various concentrations of the aqueous *C. collinum* leaves extract (CCL). Only at higher extract concentrations, the metabolic activity of the HaCaT cells decreased. The IC_50_ value of the reduction of metabolic activity was calculated with 1005 µg/mL (Supplementary Material). Subsequently, the selectivity index was calculated from the IC_50_ values from the MTT test and anti-inflammatory tests. The selectivity index of the CCL extract for IL-8 was 7.05 for IL-8 and 1.85 for IL-6, respectively.

**Figure 5:**
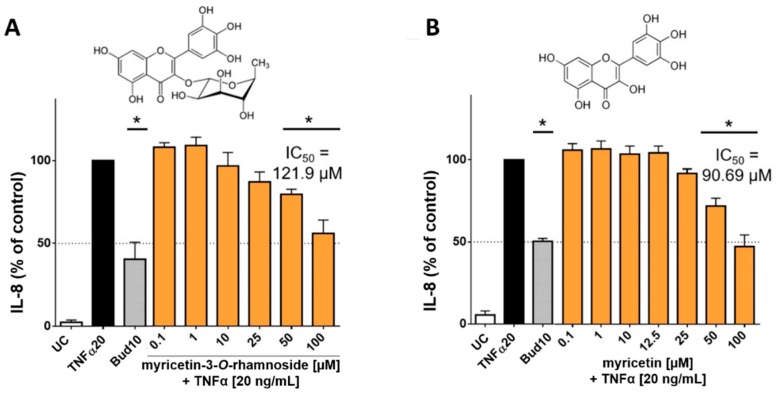
Influence of myricetin-3-*O*-rhamnoside and myricetin on IL-8 released by HaCaT cells after stimulation with TNFα (20 ng/mL): The main phenolic compound of CCL, myricetin-3-*O*-rhamnoside (**A**) and its aglycone myricetin (**B**) concentration-dependently reduced the levels of IL-8 at nontoxic concentrations. The compounds were tested in concentrations ranging from 0.1–100 µM, resulting in IC_50_ values for IL-8 of 121.9 µM for myricetin-3-*O*-rhamnoside (**A**) and 90.69 µM for myricetin (**B**), respectively. 10 µM budesonide (Bud 10) was used as a positive control, UC = Untreated control, *n* = 4–6 replicates, data presented as mean ± SEM, significant for * *p* ≤ 0.05 compared to TNFα control in ordinary one-way ANOVA.

In order to investigate whether the main flavonoids of CCL are responsible for reducing the release of interleukins, additional tests were performed with pure flavonoid substances. The phytochemical studies had confirmed myricetin-3-*O*-rhamnoside as the main constituent along with several other glycosylated myricetin derivatives. During the ethnomedical application, cleavage of the aglycon can be caused by thermal treatment (boiling or roasting) [18], or by other components with β-glucosidase activity in herbal mixtures [19]. During application to skin tissue, flavonoid glycosides may also be susceptible to enzymatic hydrolysis [20]. Therefore, not only myricetin-3-*O*-rhamnoside was tested, but also myricetin to determine the effect of the aglycone. Hence, HaCaT cells were treated with TNFα (20 ng/mL) in co-incubation with selected flavonoids, which were applied in concentrations of 0.1–100 µM. The applied concentrations of myricetin-3-*O*-rhamnoside corresponded to the concentration of myricetin-3-*O*-rhamnoside in the CCL extract, which was determined by HPLC (12.72% ± 1.40% of myricetin-3-*O*-rhamnoside in the CCL extract). There was only a minor influence on metabolic activity (data not shown). The evaluation of IL-8 in the supernatants of cells incubated with TNFα and myricetin-3-*O*-rhamnoside revealed a reduction of IL-8 levels comparable to the total extract at 50 µM and 100 µM, resulting in a calculated IC_50_ value of 121.9 µM (Figure 5A). However, a reduction of IL-6 levels was not detected for myricetin-3-*O*-rhamnoside (data not shown).

Tests with the aglycone myricetin yielded similar results. The upregulation of IL-8 was inhibited by myricetin in concentrations between 50 to 100 µM resulting in an IC_50_ value of 90.69 µM (Figure 5B). An effect for IL-6 could not be detected in our model for myricetin. The lower IC_50_ value of myricetin in comparison to myricetin-3-*O*-rhamnoside in influencing IL-8 release can be well explained by the higher lipophilicity of the aglycone. The molecule thus possesses a better ability to penetrate into the cell interior of the keratinocytes in order to cause an effect [21]. Our findings are in good agreement with previous studies, which have already found anti-inflammatory effects for myricetin and its glycosylated derivatives, including myricetin-3-*O*-rhamnoside [22,23]. The effect of myricetin and its derivatives appears to be related to the neutralization of reactive oxygen species, which is also highlighted by the antioxidant effect of the extract and its major components [22]. The mechanism of action of the anti-inflammatory effect of flavonoids is probably due to a blockage of the NFkB pathways [24]. IL-8 is a key mediator in acute inflammation and plays a central role in the attraction of neutrophils and the degranulation of neutrophils [25]. Increased levels of IL-8 have been repeatedly found in inflammatory skin diseases [26]. IL-6 is generally regarded as a proinflammatory cytokine, but it is now accepted that it also has both regenerative and anti-inflammatory properties [27]. Our results show that both *C. collinum* leaves extract and the pure substances myricetin-3-*O*-rhamnoside and myricetin attenuate the IL-8 response in TNFα stimulated HaCaT cells, thus suggesting a moderate anti-inflammatory effect. The slight reduction of IL-6 by CCL, however, must be caused by other compounds, since myricetin-3-*O*-rhamnoside and myricetin did not show any effect on this cytokine.

### 2.4. In-Vitro Antibacterial Activity of C. collinum EtOH 50% Leaves Extract

*Combretum collinum* ethanolic leaves (CCL EtOH 50%) extract was investigated for its antibacterial effect against *S. aureus* and *S. epidermidis* as well as *MRSA* since it is used against bacterially infected wounds of the skin in African traditional medicine [5]. *Klebsiella pneumoniae* was tested due to the ethnomedical application of *C. collinum* against diseases of the upper respiratory tract [6]. Since the plant is used against dysentery and diarrhea, *E. coli* and *Enterococcus sp*. were also tested [6]. According to our findings, no further published results on the antibacterial activity of *C. collinum* from Benin are available so far. The tests with a stock solution in the agar well diffusion assay at a concentration of 10 mg/mL showed that the extract was potentially effective against *S. epidermidis, S. aureus*, *MRSA* as well as *K. pneumoniae. Enterococcus* spec. and *C. albicans* were resistant against the extract. In addition, only a low activity against *P. aeruginosa* and *E. coli* was observed. In the agar well diffusion assay, the strongest effect was demonstrated against *Staphylococcus* species with *S. epidermidis* and *MRSA* exhibiting the greatest sensitivity to the extract (Table 4).

The two most sensitive strains, *S. epidermidis* and *MRSA* were subsequently selected and the minimum inhibitory concentration of the extract against these bacteria was determined. MIC values resulted in 275.0 µg/mL for *S. epidermidis* and 385.5 µg/mL for *MRSA*, respectively (Table 4).

**Figure 6:**
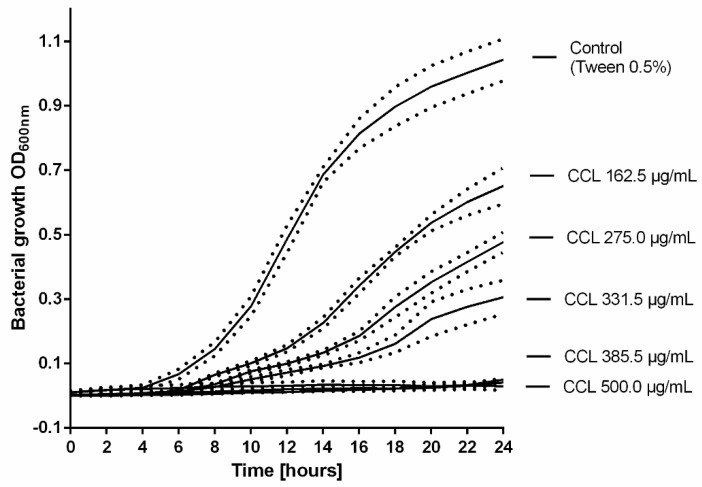
The growth profile of *MRSA* (ATCC 43300) treated with increasing concentrations of *C. collinum* ethanolic leaves extract (CCL EtOH 50%). The results are plotted as means of three experiments ± SEM (dotted line), the control was the solvent 0.5% Tween 80.

Subsequently, the growth profile of *MRSA* (ATCC 43300) treated with increasing concentrations of CCL EtOH 50% was recorded (Figure 6). As shown in Figure 6, the growth curves displayed a concentration-dependent inhibition of *MRSA* growth under co-incubation with increasing concentrations of CCL EtOH 50%. Complete inhibition of bacterial growth was achieved with 385.5 µg/mL of CCL EtOH 50. The solvent control 0.5% Tween 80 had no effect on bacterial growth.

The antimicrobial activity against *MRSA* is higher compared to a great number of other reports of ethanolic plant extracts with phenolic constituents whose MICs often exceed 1 mg/mL [28,29,30]. However, according to Cos et al. [31], who suggested a MIC of at least 100 µg/mL to substantiate pronounced antimicrobial activity of natural products, our results support only a moderate antimicrobial effect against species of *Staphylococcus* that play an important role in the infection of skin wounds. These results are in agreement with the findings of de Morais Lima et al. [32], who reported the effects of other species of *Combretum* against *Staphylococcus* species. An effect against *Pseudomonas* species demonstrated in previous studies for extracts of the aboveground parts of *C. collinum,* could not be confirmed. The variation could be due to different extraction methods and the bacterial strains used. In general, many studies have already demonstrated the efficacy of plant extracts and their respective compounds in blocking microbial growth [33]. With regard to our phytochemical investigations, it can be assumed that the polyphenols representing the main components of the extract could contribute to the antimicrobial effects of the extract. Polyphenols, in particular, have an influence on the activity of bacterial enzymes and proteins and on the fluidity of bacterial cell membrane [34]. Thus, they can alter the proton gradient or the ion balance at the cell membrane, which ultimately leads to bacterial cell death [35]. The activity against *Staphylococcus* species can presumably be explained by the influence on multiple virulence factors, which previous studies have already demonstrated for polyphenols [36]. For example, myricetin could effectively inhibit *S. aureus* biofilm formation at a concentration of 1 µg/mL [36]. Glycosylated myricetin derivatives such as myricetin-3-*O*-rhamnoside tend to show higher MIC values of up to 250 µg/mL [37].

## 3. Materials and Methods

### 3.1. Plant Material

Leaves of *Combretum collinum,* Fresen (*Combretaceae*) were collected in December 2015 in Basila, in the department Donga, Benin. The corresponding geographical coordinates were 8.57°N, 1.39°W. The plant material was identified by botanist Pierre Agbani (University of Abomey-Calavi). An herbarium specimen was deposited at the Herbarium in Leipzig with ID number LZ 225206.

### 3.2. Phytochemical Analysis

#### 3.2.1. GC-MS analysis of Hexane Extract

Thirty g of dried *C. collinum* leaves were extracted by Soxhlet extraction with 600 mL of *n*-hexane (HPLC-Quality, Roth, Karlsruhe, Germany) for 6 h. The resulting extract was evaporated to dryness to yield 1.008 g of dry extract (corresponding to 3.39%). Derivatization of the extract (168 µL of 1 mg/mL hexane extract solution corresponding to 5 mg dry mass of *C. collinum* leaves) was performed after adding 60 µL *N*,*O*-bis(trimethylsilyl)trifluoroacetamide (BSTFA, Supelco Inc., Bellefonte, PA, USA) and incubation of the mixture in a sealed vial at 60 °C for 2 h. An amount of 0.2 µL and 1 µL of the silylated mixture was analyzed by gas chromatography-mass spectrometry (GC-MS) in a 6890 N GC System with Agilent 5973 mass selective detector, single quad (MSD, Agilent Technologies, Santa Clara, CA, USA) as follows: Mobile phase: He (flow 0.9 mL/min); stationary phase: DB-35MS UI (30 m × 0.25 mm × 0.25 µm) (Agilent Techn., Böblingen, Germany); Inlet: 250 °C, splitless; temperature gradient: 100 °C (3 min, isothermal) to 250 °C (12 °C/min), to 300 °C (3 °C/min), 300 °C, 4 min isothermal; EI at 70 eV; SCAN mode; scan range *m*/*z* 50–750. Acquired data was analyzed by MSD ChemStation version F.01.01.2317 (Agilent Technologies, Waldbronn, Germany) and the MS-spectra of the chromatographic peaks were identified by search against the NIST08 database (National Institute of Standards and Technology, Gaithersburg, MD, USA). A detailed manual verification of the assigned structures was performed, also taking into consideration information on retention behaviour and fragmentation pattern. In addition, the identity of several standards was confirmed by co-spiking experiments.

#### 3.2.2. Chromatographic Separation and Isolation of Flavonoids

The isolation and characterization of the components of the leaves of *C. collinum* was achieved by applying a modified isolation method described by Kisseih et al. [12]. Pulverized plant material (600 g) was degreased by Soxhlet extraction in several batches of 150 g each with 600 mL petroleum ether (Roth) over a period of 7 h. The resulting extracts were combined and evaporated under vacuum (m = 18.65 g; corresponding to 3.09%). The remaining solvent was evaporated overnight under a hood, and the degreased material was dried and then reweighed. In the following step, the degreased plant material was extracted in portions of 100 g in 1 L 50% ethanol (EtOH) for 5 min with a rotor-stator extractor (UltraTurrax, Ika, Staufen, Germany) at 6.000 rpm each. The extract was filtered and evaporated under vacuum at 40 °C and then lyophilized (yield: m = 82.91 g; corresponding to 14.1% related to the degreased plant material). 15 g of the obtained dry extract was dissolved in water and partitioned against ethyl acetate (EtOAc, p.a., quality, Roth) eight times. The united aqueous and ethyl acetate phases from all partitions were evaporated to dryness under reduced pressure and then lyophilized (EtOAc phase: yield 13.9% related to EtOH 50% extract/aqueous phase: 44% related to EtOH 50% extract). 1 g of the ethyl acetate extract was then separated on 45 g Sephadex™ LH-20 (Sigma-Aldrich, Taufkirchen, Germany) column (3.5 cm diameter and height 25 cm height) with ethanol 96% (p.a., Roth) and 60 fractions were collected. The individual fractions were investigated for their chromatographic profile on TLC plates (Silica Gel 60 F 254, Macherey-Nagel, Düren, Germany) using water: formic acid: ethyl acetate (5:5:90, *v*/*v*) as solvent. The detection was carried out in UV 254 nm and UV 365 nm, as well as after spraying with natural product reagent A (1% diphenylboric acid 2-aminoethyl ester in methanol) and Macrogol 400 (5% in methanol) as well as 0.4 mM 2,2-diphenyl-1-picrylhydrazyl radical (DPPH) in methanol. In the following, fractions were selected on the basis of their chromatographic profile and subjected to HPLC analysis in order to decide which fractions could be usefully combined. Selected fractions were then subjected to further analytical methods (liquid chromatography-mass spectrometry (LC-MS), nuclear magnetic resonance (NMR)). Compound **2** was obtained in a quantity of 42.9 mg from the pooled fractions F 31–F 33 obtained by precipitation in ethyl acetate.

#### 3.2.3. HPLC Analysis

High-performance liquid chromatography (HPLC) analysis was used to study the fingerprint profile of the EtOH 50% extract of the dried leaves of *C. collinum*, for spiking experiments to further confirm the identity of individual substances and to check the purity of the fractions obtained after Sephadex column chromatography. To analyze the fingerprint profile, a solution of 5 mg/mL of the EtOH 50% extract of the dried leaves of *C. collinum* was prepared in 5% acetonitrile/water (ACN/H_2_O). Subsequently, the solution was centrifuged at 8000 *g* for 10 min to remove any suspended particles. For spiking experiments to confirm individual substances, the reference compounds myricetin-3-*O*-rhamnoside and myricetin-3-*O*-glucoside (Extrasynthese, Genay, France) were dissolved in methanol. All HPLC experiments were performed on an analytical column from Macherey-Nagel (MN) EC 250/4; Nucleosil 100-5 C18 with a pump and column oven (Dionex UltiMate 3000 compartments, Thermo Scientific, Dreieich, Germany) connected to a Waters 717plus autosampler and Waters 996 Photodiode Array Detector (Waters, Eschborn, Germany). The injected sample volume was 10 µL 0.1% aqueous formic acid and 0.1% formic acid in acetonitrile were used as solvents A and B, respectively, at a flow rate of 1 mL/min at 25 °C with the following gradient: *t*_0 min_ A 97%, *t*_30 min_ A 85%, *t*_45 min_ A 75%, *t*_50 min_ A 50%, *t*_55 min_ A 5%, *t*_60 min_ A 5%, *t*_65 min_ A 97%. To check the purity of the fractions after Sephadex fractionation, 100 µL of the fractions was centrifuged at 8000 *g* for 10 min to remove any suspended particles and then analyzed by HPLC as described as above. For all experiments, UV-Vis spectra were recorded from 220 to 400 nm. Data analysis and processing were done with the Chromeleon software version Client 6.80 (Thermo Scientific).

#### 3.2.4. Quantitative HPLC Analysis

For the quantification of myricetin-3-*O*-rhamnoside in CCL EtOH 50% and CCL aqueous extract, a stock solution of both extracts in DMSO at a concentration of 5 mg/mL was prepared. In addition, a 1 mg/mL stock solution of the standard myricetin-3-*O*-rhamnoside and serial dilutions of 700 µg/mL to 100 µg/mL were prepared. The quantification of myricetin-3-*O*-rhamnoside and myricetin-3-*O*-glucoside was then performed by HPLC with three independent injections of each extract at 350 nm with the gradient described in Section 3.2.3. The content of myricetin-3-*O*-glucoside was calculated based on the obtained calibration curve of the standard myricetin-3-*O*-rhamnoside.

#### 3.2.5. LC-MS Analysis

LC-MS experiments were performed using a mass spectrometer Esquire 3000 plus from Bruker Daltonics (Bremen, Germany). The injection volume was set to 10 µL. The iontrap instrument was equipped with an electrospray ionization source operated in the positive mode (ICC: 20,000) and negative mode (ICC: 10,000), nebulizer pressure 70 psi, dry gas 12 L/min (both nitrogen) at 365 °C and the target mass set to *m*/*z* 600. Separation of 25 µL extract was achieved on an Agilent 1100 series HPLC system operated by ChemStation Rev.B.01.03. using an eluent flow of 0.5 mL/min with the same column and gradient described in Section 3.2.3.

#### 3.2.6. NMR Experiments

NMR spectra were recorded in CD_3_OD on a Bruker MSL 500 NMR-spectrometer. Data analysis was performed in MestReNova-11.0.3 and the ^1^H-NMR spectra were compared with the data of known compounds from literature.

#### 3.2.7. DPPH Assay

Radical scavenging activity of the DPPH radical extract was determined using a modified version of the Brand-Williams method [38]. 10 μL methanolic solution of diluted samples or Trolox (Sigma-Aldrich, Taufkirchen, Germany) solution in concentrations of 100 μM to 1.56μM were added to 190 μL of DPPH solution (Sigma-Aldrich, Taufkirchen, Germany) in a 96-well plate. The final concentration of DPPH was 0.1 mM in methanol. The solution was mixed gently and incubated for 30 min at room temperature in the dark. Subsequently, the absorbance against methanol was determined with a microplate reader at 517 nm (BioTek Synergy 2 Multi-Detection Microplate Reader, BioTek Instruments Inc., Winooski, Vt, USA). The DPPH radical scavenging activity of the extracts was calculated from the standard curve of Trolox and expressed as micromoles of Trolox Equivalents (TE) per gram of sample (μmol TE/g). Solvent controls containing only 10 μL methanol instead of samples and a positive control containing Trolox were used. Pure methanol was used as a blank. The inhibition of the DPPH radical by the samples was calculated according to the following formula:

$$DPPH\;\mathrm{scavenging}\;\mathrm{activity}\;\%=\left(1-\frac{A_{sample}-A_{blank}}{A_{control}-A_{blank}}\right)\times 100\left(1-\frac{A_{\mathrm{sample}}-A_{\mathrm{blank}}}{A_{\mathrm{control}}-A_{\mathrm{blank}}}\right)$$

#### 3.2.8. ABTS Assay

This assay was performed as described by Zou et al. [39] with slight modifications. 2,2′-azino-bis(3-ethylbenzothiazoline-6-sulphonic acid) radical (ABTS) cations were obtained by adding an equal volume of 7 mM ABTS (Applichem, Darmstadt, Germany) in water to 4.9 mM potassium persulfate in water. The solution was incubated at room temperature in the dark for 10–12 h, then filtered and diluted with 80% ethanol to an absorbance of 0.70 at 734 nm. Ten µL of diluted samples was added to 190 μL of ABTS solution in a 96-well plate, and absorbance was measured at 734 nm after an incubation time of 30 min at room temperature in the dark. Trolox was used as a standard and positive control, and a standard calibration curve was obtained for Trolox ranging from concentrations of 1.56 μM to 100 μM. The Trolox Equivalent Antioxidant Capacity (TEAC) values of the samples were calculated from the Trolox standard curve and presented as Trolox equivalents in micromoles per gram of sample (μmol TE/g).

### 3.3. Functional Investigations on HaCaT Cells

#### 3.3.1. Material

An aqueous extract from the plant material was used for functional investigations. To prepare the water extracts of the dried leaves of *C. collinum*, 5 g of the powdered dried plant material was mixed with 50 mL water and treated in an ultrasonic bath for 15 min. The resulting suspension was centrifuged at 3500 rcf and the clear supernatant was removed afterwards. This step was repeated four times and the supernatants were combined and lyophilized. The residue was 565 mg thus 11.3% of the plant material. The extract was then solubilized in sterile water, sterile-filtrated and stored at −20 °C.

#### 3.3.2. Cell Culture

Immortalized human keratinocyte cells (HaCaT) were kindly provided by Dr. Ulf Anderegg, Research Laboratory of the Clinic for Dermatology, Venerology and Allergology (University of Leipzig, Germany). Immortalized HaCaT cells were cultured in Dulbecco’s Modified Eagles Medium (DMEM), (Biowest, Nuaillé, France) supplemented with 1% PenStrep (Biowest), 10% fetal calf serum (FCS, Biowest) at 37 °C in a humidified atmosphere at 5% CO_2_ in cell culture flasks (Sarstedt Nümbrecht, Germany). The experiments were routinely conducted at 80–90% confluence [40].

#### 3.3.3. In-Vitro Anti-Inflammatory Assay

The experiments were carried according to Wedler et al. [41] with some modifications. HaCaT cells were seeded in 24-well plates at a density of 5.0 × 10^4^ cells per well and incubated in the wells under the same culture conditions, as mentioned above for 24 h. After 24 h the medium was changed and the cells were incubated with medium containing different extract concentrations (1–200 µg/mL), myricetin-3-*O*-rhamnoside (0.1–200 µM), myricetin (0.1–200 µM) or the positive control containing 10 µM budesonide or the solvent control 0.125% DMSO with addition of TNFα (20 ng/mL). After an additional incubation period of 24 h with TNFα (20 ng/mL) (Humanzyme, Chicago, IL, USA), cell-free supernatants were collected. Subsequently IL-8 and IL-6 release was determined in the supernatants using ELISA kits (BD Biosciences, San Diego, CA, USA) in accordance with the manufacturer’s instructions. Absorption was measured at 450 nm with a microplate reader (Infinite M 200, TecanGroup Ltd., Mannedorf, Switzerland).

#### 3.3.4. Cell Viability Assay

In addition, cytotoxicity controls were performed on the treated HaCaT cells to confirm stable cell viability throughout the assay. For this, a 3-(4,5-dimethylthiazol-2-yl)-2,5-diphenyltetrazolium (MTT) assay was used to determine the relative amount of viable cells capable of converting MTT (Sigma-Aldrich, Taufkirchen, Germany) into the purple formazan. Briefly, after HaCaT cells were stimulated with TNFα, the supernatant was collected, and remaining cells were treated with 500 μL MTT (0.3 mg/mL in PBS) per well for 2 h. Control cells were treated with DMEM + FCS or DMEM + FCS + DMSO. Subsequently, the living cells were lysed by adding 500 μL SDS lysis buffer (20% SDS in 40% dimethylformamide) and the amount of purple formazan was determined spectrophotometrically with a microplate reader (Tecan Infinite M 200) at 570 nm and 630 nm as reference wavelength.

### 3.4. Antimicrobial Assays

#### 3.4.1. Microbial Strains

The antimicrobial activity was evaluated using the following strains: *Klebsiella pneumoniae* (ATCC 13883), *Escherichia coli* (DSM 11250), *Enterococcus spec., Pseudomonas aeruginosa* (DSM 50071), *Staphylococcus aureus* (DSM 346), *Staphylococcus epidermidis* (DSM 20044), Methicillin-resistant *Staphylococcus aureus* (*MRSA*, ATCC 43300) and *Candida albicans* (DSM 1386). The strains were provided by the Fraunhofer Institute for Cell Therapy and Immunology, Antimicrobial Agents Unit (Leipzig, Germany).

#### 3.4.2. Agar Well Diffusion Test

A modified protocol of the European Committee for Antimicrobial Susceptibility Testing (EUCAST) [42] was used for the test. Different microbial strains were first cultivated at 37 °C in an incubator for 16–20 h on CASO agar plate (3.5% casein-soya, 0.3% yeast extract, 0.1% glucose, 1.5% agar). From the microbial strains in pure, fresh culture (18–24 h), inoculum was prepared in 1 mL sterile PBS for each strain. The 0.5 McFarland solution (1–2 × 10^8^ CFU/mL) served as the standard for the preparation of microbial inoculants. The microbial inoculum was uniformly spread using a sterile cotton swab on a sterile Petri dish with Mueller-Hinton (MH) agar or Sabouraud Agar. 150 µL of extract solution containing CCL EtOH 50% diluted in 10% dimethylsulfoxide (DMSO; Sigma-Aldrich, Taufkirchen, Germany) was added to each of the wells (7 mm diameter holes cut in the agar gel, 20 mm apart from each other). Then, the plates were incubated under aerobic conditions for 24 h at 36 °C ± 1 °C. After incubation, confluent microbial growth was observed. Inhibition zones of the microbial growth were measured in mm. References gentamicin, vancomycin and nystatin were used as positive controls. 10% DMSO served as a negative control. Tests were performed in triplicate.

#### 3.4.3. Microdilution Test

Minimum inhibitory concentrations (MIC) were determined to assess the susceptibility of pathogenic bacteria to ethanolic extracts of the plants. For this purpose, extracts were dissolved in 50% Tween 80 in order to increase solubility and prevent precipitation. Subsequently, 75 μL of each bacterial suspension (corresponding to a bacterial density of 1–2 × 10^6^ CFU/mL) was pipetted into wells of microtiter plates (Greiner Bio-One GmbH, Kremsmünster, Austria) containing 75 μL of extract solution diluted in MH medium. Final concentrations of plant extract ranged from 50–500 µg/mL. After 24 h incubation at 37 °C, bacterial growth was evaluated by manual observation. The MIC was assessed as the dilution step in which no turbidity of the test dilution was observed. The negative control was pure MH medium; MH medium with the same amount of bacterial suspension served as growth control. Medium with 0.5% Tween 80 served as solvent control. Tests were performed in triplicate.

#### 3.4.4. Bacterial Growth Curve

For measuring the growth profile of *MRSA* treated with *C. collinum* ethanolic extract, extract stock solutions were prepared and pipetted into a 96-well plate as described in 3.4.3.. The plate was incubated then at 37 °C with continuous shaking at a frequency of 493 cpm (4 mm) in linear mode. The growth of *MRSA* in MH-medium was determined by measuring the optical density at 600 nm at regular intervals of 15 min using an Epoch 2 microplate spectrophotometer (BioTek, Winooski, VT, United States) over a period of 24 h. The growth curves of each concentration were plotted and compared with the profile of the solvent control (0.5% Tween).

### 3.5. Data Analysis

Spectrophotometric data from antioxidant experiments, interleukin release experiments, metabolic activity experiments and antimicrobial experiments were processed with Microsoft Excel 2013 and graphically visualized with GraphPadPrism6 (6.0, GraphPad Software Inc., GraphPad Software Inc., San Diego, Ca., USA). For statistical analysis of antioxidant experiments with *n* ≥ 3, the control was set to 100% and tested for significance against samples and positive control with GraphPadPrism6 using an ordinary one-way variance analysis (ANOVA), followed by a Tukey‘s multiple comparison test. When comparing the antioxidant activity of the extract with the antioxidant potency of the myricetin derivatives, the same statistical procedure was applied. Regarding the in vitro anti-inflammatory experiments, the observed protein release and metabolic activity were normalized to the TNFα stimulated untreated control set to 100%. The statistical analysis with GraphPadPrism6 was performed with an ordinary one-way ANOVA, followed by a Tukey‘s multiple comparison test. The maximum inhibition was then derived from the normalized values with concentration-response curves based on nonlinear regression. The concentration-response curves also yielded the half-maximum inhibition concentrations (IC_50_).

## 4. Conclusions

The phytochemical studies revealed myricetin-3-*O*-rhamnoside and myricetin-3-*O*-glucoside as the main compounds of the hydrophilic extracts. Both substances showed antioxidant activity and contribute significantly to the overall antioxidant activity of the extracts. In the present study, we provided evidence that the aqueous extract of the leaves of *C. collinum* has an in vitro anti-inflammatory effect in TNFα stimulated HaCaT cells. This supports the benefit of ethnomedical use in various inflammatory diseases in traditional African medicine. Further investigations of the main components showed that the main compound myricetin-3-*O*-rhamnoside also exhibits an in vitro anti-inflammatory effect, but only on IL-8 secretion. The comparison of the inhibition of IL-8 release by myricetin-3-*O*-rhamnoside and myricetin showed that the aglycone seems to be responsible for a major part of the effect. The antimicrobial activity of *C. collinum* leaves extract was most effective against *Staphylococcus* species, which play a major role in bacterially infected wounds of the skin. This validates -to some extent- the traditional medicinal use of *C. collinum* for this purpose. Consequently, the ethnomedical application of *C. collinum* leaf extracts in topical inflammations and infections can be supported by the results of these investigations and thus potentially represents an alternative treatment strategy for inflammatory skin diseases and related infections.

## Figures and Tables

**Table 1:** Summary of chromatographic, UV-Vis-spectroscopic and mass spectrometric data of the main compounds **1** and **2** of the EtOH 50% extract of *C. collinum.*

Peak	RT ^1^ [min]	*λ*_max_ [nm]	*m*/*z*	Ion	Compound
1	20.177	264 350	319.096 481.189 479.193	[A + H]^+^ [M + H]^+^ [M − H]^−^	Myricetin-3-*O*-glucoside
2	23.219	262 350	319.094 465.179 463.188	[A + H]^+^ [M + H]^+^ [M − H]^−^	Myricetin-3-*O*-rhamnoside

^1^ RT = Retention time.

**Table 2:** Chemical shifts (^1^H (ppm), 350 MHz, methanol-*d*_4_) and measurement conditions of the isolated myricetin-3-*O*-rhamnoside compared to data from literature [11].

Signal	Myricetin-3-*O*-rhamnoside (F31–F33)	Myricetin-3-*O*-rhamnoside [10]
H-3′′-6′′	0.95, d, *J* 6 Hz	0.94, d, *J* 5.6 Hz
sugar H	3.3–3.8	3.3–3.8
H-2′′	4.25, s	4.23, s
H-1′′	5.34, brs	5.31, brs
H-6	6.22, s	6.19, s
H-8	6.39, s	6.35, s
H-2′ and H-6′	6.97, s	6.95, s

**Table 3:** Summary of the compounds identified from the *n*-hexane extract of the leaves of *C. collinum* after silylation with *N*,*O*-bistrifluoroacetamide (BSTFA) by NIST08 database. Substances in bold were confirmed by spiking experiments with reference compounds. Number labels correspond to the compounds listed in Figure 2.

No.	RT ^1^ [min]	Compound	NIST Match Quality
1	12.62	Malic acid	97
2	12.73	Tetradecanoic acid (syn. myristic acid)	98
3	14.39	Hexadecanoic acid (syn. palmitic acid)	98
4	15.87	Oleic acid	98
5	15.92	Octadecanoic acid (syn. stearic acid)	99
6	17.64	Eicosanoic acid (syn. arachidic acid)	99
7	19.78	Docosanoic acid (syn. behenic acid)	99
8	22.40	Tetracosanoic acid (syn. lignoceric acid)	99
9	17.94	Pentacosane	96
10	18.99	Hexacosane	99
11	20.17	Heptacosane	99
12	21.48	Octacosane	99
13	22.92	Nonacosane	96
14	24.42	Triacontane	99
15	26.06	Nonadecane	96
16	23.22	Squalene	97
17	30.78	Campestrol	99
18	31.18	Stigmasterol	99

^1^ RT = Retention time.

**Table 4:** Antimicrobial screening of *C. collinum* ethanolic leaves extract (CCL EtOH 50%, 10 mg/mL) and positive controls.

Species	Inhibition Zones (mm)	Inhibition Zones (Positive Controls)	MIC
***S. epidermidis***	**21.75 ± 0.43**	28.67 ± 0.47 (G)	**275.0 µg/mL**
***MRSA***	**16.25 ± 0.43**	18.67 ± 0.47 (V)	**385.5 µg/mL**
***S. aureus***	**15.75 ± 0.43**	24.33 ± 0.47 (G)	n.d.
*K. pneumoniae*	14.00 ± 0.00	25.33 ± 0.94 (G)	n.d.
*P. aeruginosa*	11.00 ± 0.00	12.00 ± 0.00 (G)	n.d.
*E. coli*	11.25 ± 0.43	26.67 ± 0.47 (G)	n.d.
*Enterococcus*	0	16.67 ± 0.47 (G)	n.d.
*C. albicans*	0	26.67 ± 0.47 (N)	n.d.
DMSO 10%	0	-	n.d.

(G—gentamicin 10 µg, V—vancomycin 5 µg, N—nystatin 50 µg) against some bacterial strains and *C. albicans*. In addition, the MIC values of *S. epidermidis* and *MRSA* are given. *Staphylococcus* species showed the highest sensitivity towards the extract (values given in bold). n.d.—not determined.

## Supplementary Materials

The following are available online, Figure S1. Representative HPLC chromatogram of the EtOH 50% extract (A) and the EtOAc extract (B) of *C. collinum* (5 mg/mL extract dissolved in ACN 5%, injection volume 20 µL, detection wavelength = 320 nm). The molar mass of the main compounds could be confirmed by LC-MS experiments. For compound **1** = myricetin-3-*O*-glucoside LC-MS mass spectra in negative mode is shown in I and positive mode in II; for compound **2** = myricetin-3-*O*-rhamnoside mass spectrum in negative mode is shown in III and positive mode in IV. The compounds were further confirmed by ^1^H-NMR and spiking experiments. Manual analysis of the UV spectra of the chromatogram also yielded flavonoidal UV spectra for the smaller side peaks (marked with an asterisk in the chromatogram), with absorption maxima similar to those of myricetin derivatives (264/350 nm). In the LC-MS analysis, these peaks also showed an aglycone peak in positive mode with a mass of *m*/*z* 319, which indicates a myricetin aglycone. Figure S2. Metabolic activity of HaCaT cells after coincubation with *C. collinum* aqueous leaves extract and stimulation with TNFα (20 ng/mL): The aqueous leaves extract of *C. collinum* (CCL) did not show significant influence on metabolic activity in concentrations from 1–100 µg/mL, when 10 µM budesonide (Bud 10) was used as positive control. UC = Untreated control, N = 4–6 measurements, data presented as mean ± SEM, significant for *p* ≤ 0.05.
