# Supplementary material for: Phytochemical Characterization and In Vitro Anti-Inflammatory, Antioxidant and Antimicrobial Activity of Combretum Collinum Fresen Leaves Extracts from Benin
Source: Molecules. 2020 Jan 10;25(2):288. doi: 10.3390/molecules25020288 (PMC7024300; doi:10.3390/molecules25020288)
Supplement: Supplementary file 1 [file molecules-25-00288-s001.zip › Supplementary Files revised/Figure_S1_revised.docx]

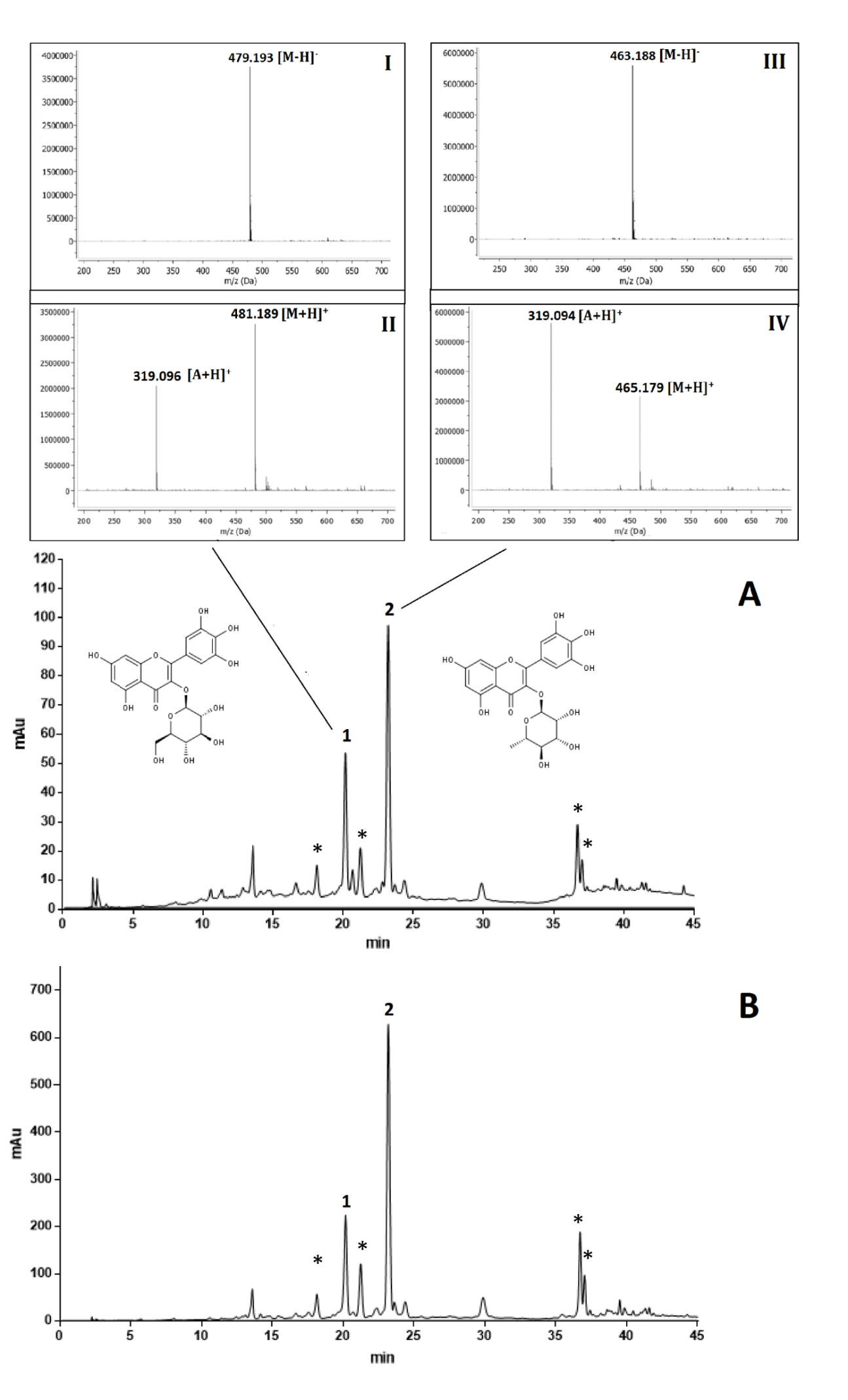


**Figure S1.** Representative HPLC chromatogram of the EtOH 50 % extract (**A**) and the EtOAc extract (**B**) of *C. collinum* (5 mg/mL extract dissolved in ACN 5%, injection volume 20 µL, detection wavelength= 320 nm). The mass of the main compounds could be confirmed by LC-MS experiments. For compound **1**= myricetin-3-*O*-glucoside LC-MS mass spectra in negative mode is shown in I and positive mode in II; for compound **2**= myricetin-3-*O*-rhamnoside mass spectrum in negative mode is shown in III and positive mode in IV, The compounds were further confirmed by ^1^H-NMR and spiking experiments. Manual analysis of the UV spectra of the chromatogram also yielded flavonoidal UV spectra for the smaller side peaks (marked with asterisk in the chromatogram), with absorption maxima similar to those of myricetin derivatives (264 nm/350 nm). In the LC-MS analysis, these peaks also showed an aglycone peak in positive mode with a mass of m/z 319, which indicates a myricetin aglycone part.
