# Supplementary material for: Phytochemical Characterization and In Vitro Anti-Inflammatory, Antioxidant and Antimicrobial Activity of Combretum Collinum Fresen Leaves Extracts from Benin
Source: Molecules. 2020 Jan 10;25(2):288. doi: 10.3390/molecules25020288 (PMC7024300; doi:10.3390/molecules25020288)
Supplement: Supplementary file 1 [file molecules-25-00288-s001.zip › Supplementary Files revised/Figure_S2_revised.docx]

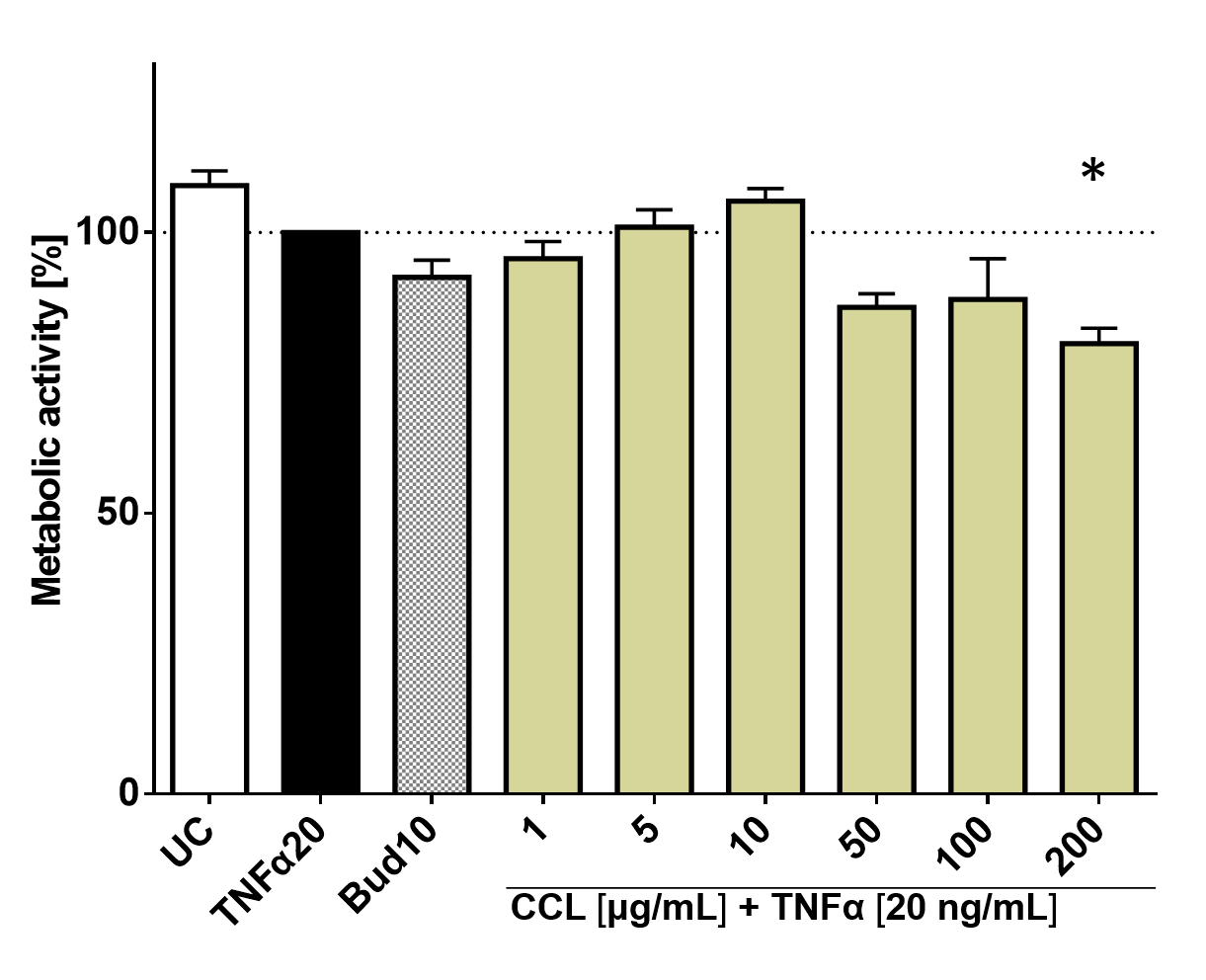


**Figure S2**: Metabolic activity of HaCaT cells after coincubation with *Combretum collinum* aqueous leaves extract and stimulation with TNFα 20 ng/mL: The aqueous leaf extract of *Combretum collinum* (CCL) did not show significant influence on metabolic activity in concentrations from 1‑100 µg/mL, Budesonide 10 µM (Bud 10) was used as positive control, UC = Untreated control, N = 4-6 measurements, data presented as mean ± SEM, significant for p ≤ 0,05.
