# Supplementary figures and images for: Phytochemical Characterization and In Vitro Anti-Inflammatory, Antioxidant and Antimicrobial Activity of Combretum Collinum Fresen Leaves Extracts from Benin
Source: Molecules. 2020 Jan 10;25(2):288. doi: 10.3390/molecules25020288 (PMC7024300; doi:10.3390/molecules25020288)

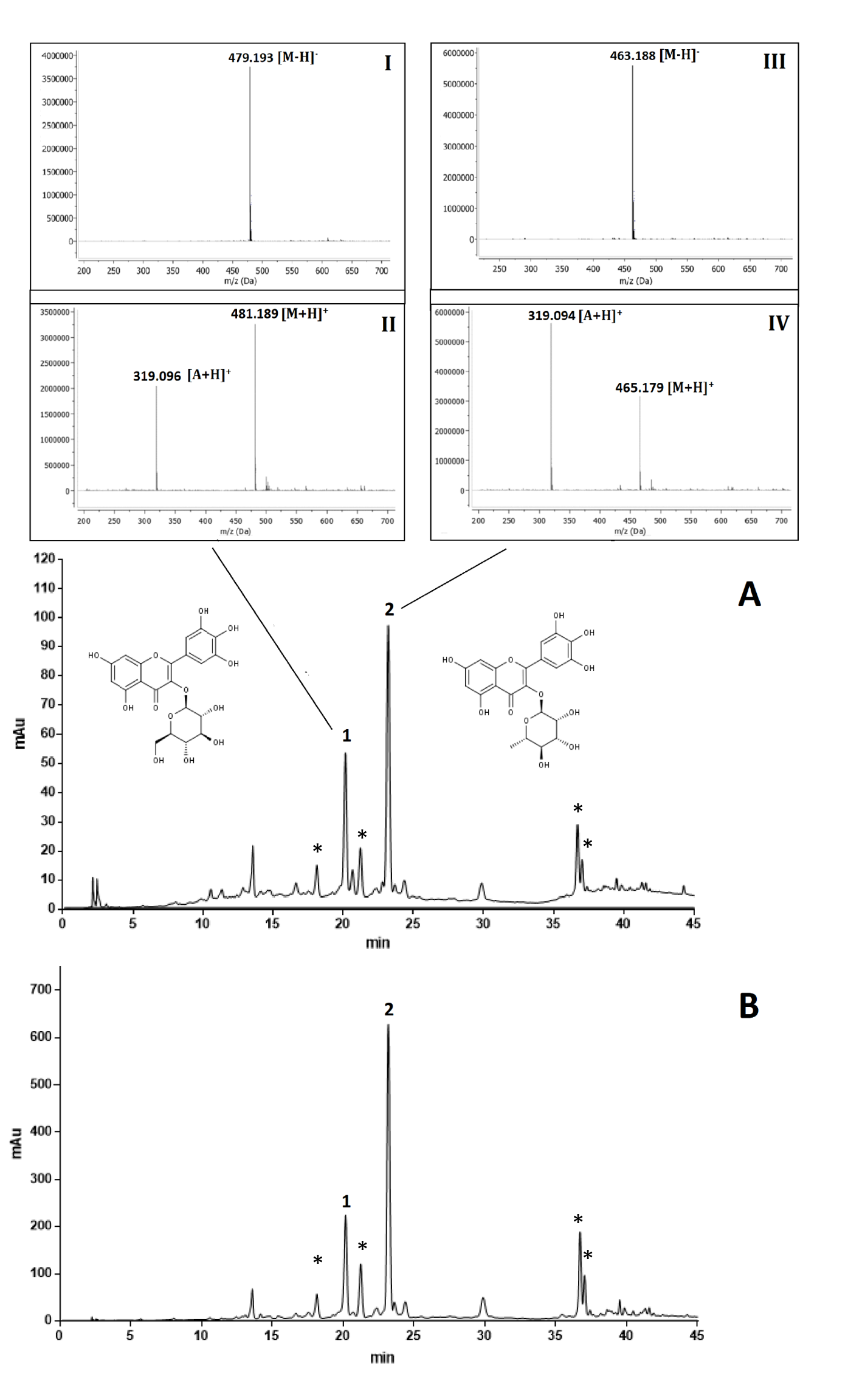

Supplement: Supplementary file 1 [file molecules-25-00288-s001.zip › Supplementary Files revised/FigureS1_revised.tif]

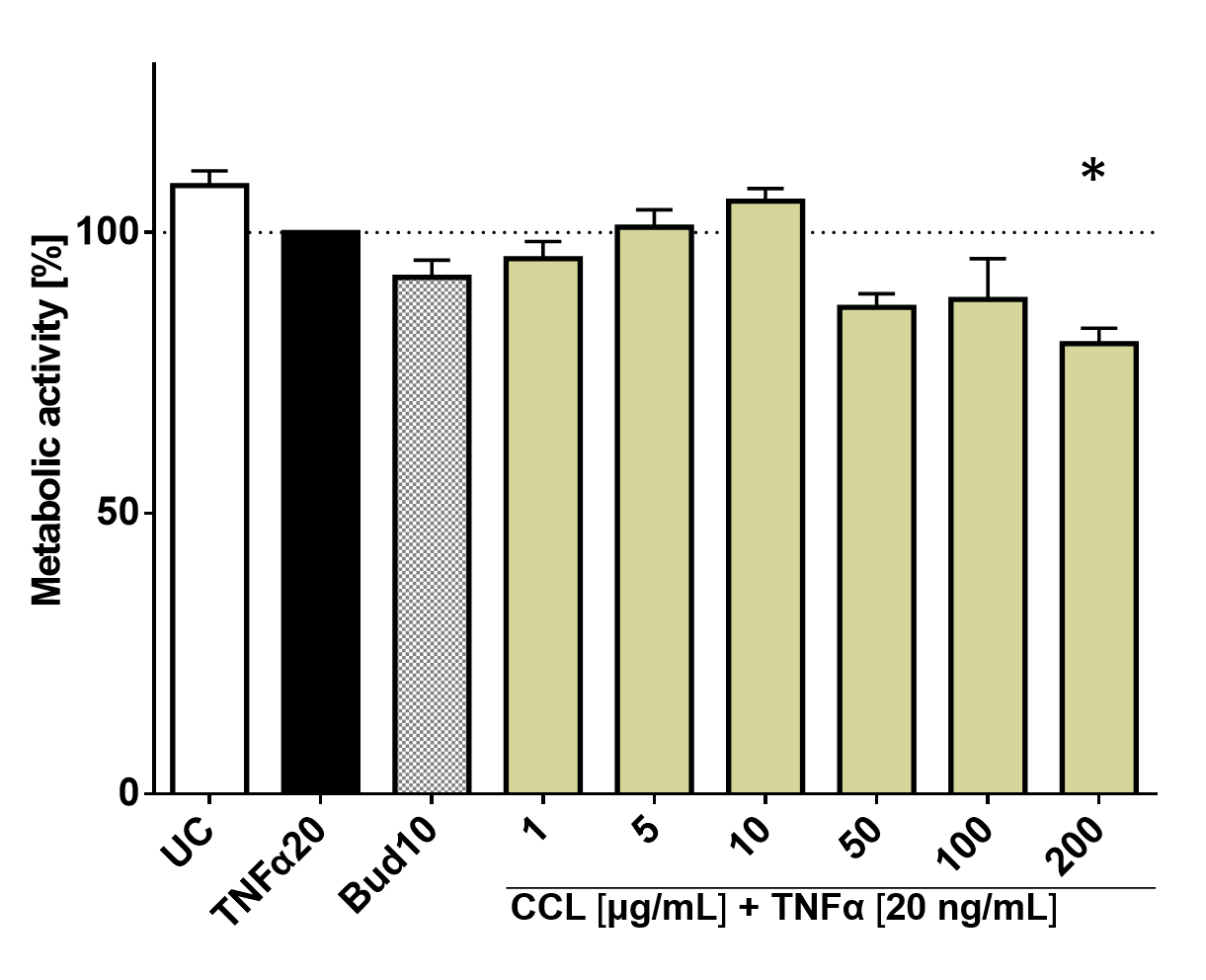

Supplement: Supplementary file 1 [file molecules-25-00288-s001.zip › Supplementary Files revised/FigureS2_revised.tif]
